# Association of serum interleukin‐27 with the exacerbation of chronic obstructive pulmonary disease

**DOI:** 10.14814/phy2.12069

**Published:** 2014-07-03

**Authors:** Takashi Angata, Takeo Ishii, Congxiao Gao, Kazuaki Ohtsubo, Shinobu Kitazume, Akihiko Gemma, Kozui Kida, Naoyuki Taniguchi

**Affiliations:** 1 Systems Glycobiology Research Group, Global Research Cluster, Wako, Saitama, Japan; 2 Institute of Biological Chemistry, Academia Sinica, Taipei, Taiwan; 3 Respiratory Care Clinic, Nippon Medical School, Tokyo, Japan; 4 Division of Pulmonary Medicine, Infectious Diseases and Oncology, Department of Internal Medicine, Nippon Medical School, Tokyo, Japan

**Keywords:** Adaptive immunity, biomarkers, inflammation, innate immunity, Siglec‐14

## Abstract

We have previously demonstrated that chronic obstructive pulmonary disease (COPD) patients who do not have Siglec‐14 are less prone to exacerbation of the disease. Siglec‐14 is a myeloid cell protein that recognizes bacteria and triggers inflammatory responses. Therefore, soluble mediators secreted by myeloid cells responding to Siglec‐14 engagement could be involved in the pathogenesis of exacerbation and could potentially be utilized as biomarkers of exacerbation. To find out, we sought genes specifically induced in Siglec‐14^+^ myeloid cells and evaluated their utility as biomarkers of COPD exacerbation. Using DNA microarray, we compared gene expression levels in Siglec‐14^+^ and control myeloid cell lines stimulated with or without nontypeable *Haemophilus influenzae* to select genes that were specifically induced in Siglec‐14^+^ cells. The expressions of several cytokine and chemokine genes were specifically induced in Siglec‐14^+^ cells. The concentrations of seven gene products were analyzed by multiplex bead array assays in paired COPD patient sera (*n* = 39) collected during exacerbation and stable disease states. Those gene products that increased during exacerbation were further tested using an independent set (*n* = 32) of paired patient sera. Serum concentration of interleukin‐27 (IL‐27) was elevated during exacerbation (discovery set: *P* = 0.0472; verification set: *P* = 0.0428; combined: *P* = 0.0104; one‐sided Wilcoxon matched‐pairs signed‐rank test), particularly in exacerbations accompanied with sputum purulence and in exacerbations lasting more than a week. We concluded that IL‐27 might be mechanistically involved in the exacerbation of COPD and could potentially serve as a systemic biomarker of exacerbation.

## Abstract (precis)

e12069

Chronic obstructive pulmonary disease (COPD) patients who do not have Siglec‐14 (a myeloid cell protein that triggers inflammatory responses) are less prone to exacerbation of the disease. We tested whether soluble mediators secreted by myeloid cells responding to Siglec‐14 engagement could be utilized as biomarkers of exacerbation, and found serum concentration of interleukin‐27 (IL‐27) was elevated during exacerbation, particularly in exacerbations accompanied with sputum purulence. We concluded that IL‐27 might be mechanistically involved in the exacerbation of COPD and could potentially serve as a systemic biomarker of exacerbation.

## Introduction

Chronic obstructive pulmonary disease (COPD) is projected to be world's third leading cause of death by 2030 (World Health Organization 2008). At present, treatment for its exacerbation accounts for much of the medical cost associated with the disease (Andersson et al. 2002; Celli and MacNee 2004; Toy et al. 2010). Exacerbation of COPD is defined as “an event in the natural course of the disease characterized by a change in the patient's baseline dyspnea, cough, and/or sputum exceeding normal day‐to‐day variations, which is acute in onset and may warrant a change in regular medication in a patient with underlying COPD” (Rabe et al. 2007). This diagnosis is based solely on clinical presentation. A quantifiable biomarker of COPD exacerbation would be useful for physicians, especially those who may not be as familiar with the disease entity as pulmonologists, so that they may make timely and objective diagnoses of exacerbation. Finding such systemic biomarkers is a formidable challenge (Koutsokera et al. 2012). One difficulty lie in the fact COPD exacerbation seemingly starts as a local rather than systemic event (Muller and Tamm 2006). One of the best systemic biomarkers used to distinguish between the stable disease and exacerbation (Hurst et al. 2006) is a systemic biomarker of acute inflammation, plasma C‐reactive protein (CRP), which is elevated in the circulation of patients with exacerbated COPD (Dev et al. 1998). One problem with plasma CRP level is that it is elevated in many pyogenic infections and thus not specific to exacerbation of COPD, and another problem is that it is not elevated in a significant proportion of patients undergoing exacerbation of COPD (Muller and Tamm 2006). Therefore, a novel systemic biomarker with genetic and mechanistic correlations to the etiology of COPD and/or its exacerbation is needed (Carter et al. 2011, 2013).

Most cases of COPD exacerbation are thought to be triggered by airway infection (Sethi and Murphy 2008). In a previous genotype–phenotype correlation study, we found that COPD patients without Siglec‐14 were less prone to exacerbation (Angata et al. 2013). Siglec‐14, a myeloid cell membrane protein, interacts with nontypeable *Haemophilus influenzae* (NTHi) and augments proinflammatory responses. In that previous study, we suggested that inflammatory responses triggered by Siglec‐14 may be involved in the exacerbation of COPD (Angata et al. 2013). In this study, we hypothesize that proinflammatory secreted mediators induced by the engagement of Siglec‐14 may be involved in its pathogenesis and they could potentially be utilized as biomarkers of that exacerbation. To find out, we identified genes induced in Siglec‐14^+^ myeloid cells by NTHi stimulation, selected gene products quantifiable in serum, and measured their concentrations in the paired sera from COPD patients collected during exacerbation and stable phases of the disease. We found IL‐27, a cytokine involved in T‐cell differentiation and regulation, to be increased in the sera of patients with COPD during exacerbation. In particular, serum IL‐27 was elevated in the exacerbations accompanied with sputum purulence and in prolonged exacerbation episodes lasting more than a week. We conclude that IL‐27 could potentially be useful as a biomarker in the diagnosis and follow‐up of COPD exacerbation.

## Materials and Methods

### Gene expression profiling of myeloid cells with or without NTHi stimulation

Siglec‐14/THP‐1 and Siglec‐5/THP‐1 cell lines (the THP‐1 sublines expressing Siglec‐14 or Siglec‐5 protein, respectively) were prepared as reported previously (Yamanaka et al. 2009). Siglec‐14/THP‐1 and Siglec‐5/THP‐1 mimic monocytes from homozygous wild‐type and homozygous *SIGLEC14*‐null people, respectively (Angata et al. 2013). These cells (5 × 10^6^ cells, suspended at 1 × 10^6^ cells/mL in Macrophage‐SFM; Life Technologies, Grand Island, NY) were incubated with or without heat‐killed NTHi strain 2019 (equivalent of 5 × 10^8^ colony forming units; cells‐to‐bacteria ratio = 1:100) for 6 h. In total, four separate samples were prepared as follows: (1) Siglec‐14/THP‐1, incubated with NTHi (designated as Siglec‐14,NTHi[+]); (2) Siglec‐14/THP‐1, incubated without NTHi (Siglec‐14,NTHi[−]); (3) Siglec‐5/THP‐1, incubated with NTHi (Siglec‐5,NTHi[+]); and (4) Siglec‐5/THP‐1, incubated without NTHi (Siglec‐5,NTHi[−]). The cells were harvested by centrifugation, and total RNA was extracted using RNeasy Mini kit (Qiagen, Hilden, Germany).

Gene expression analysis using GeneChip Human Genome U133 Plus 2.0 Array (Affymetrix, Santa Clara, CA) was performed by Dr. Keisuke Fukumoto (Support Unit for Bio‐material Analysis in RIKEN BSI Research Resources Center) following manufacturer's instructions. Briefly, 250 ng of total RNA was used for the synthesis of biotinylated RNA using GeneChip 3′ IVT Express Kit (Affymetrix). The labeled RNA was hybridized to GeneChip Human Genome U133 Plus 2.0 Array at 45°C for 16 h in GeneChip Hybridization Oven 640 (Affymetrix). The array was then washed and stained using GeneChip Hybridization, Wash, and Stain Kit (Affymetrix) on GeneChip Fluidics Station 450 (Affymetrix), and then scanned using GeneChip Scanner 3000 7G (Affymetrix). GeneChip Operating Software (Affymetrix) was used for the quantification and standardization of the signals. Signals were standardized based on the cumulative signal intensity of all probes. The gene expression data were deposited into the Gene Expression Omnibus Database (GSE47929).

Genes that fulfilled the following criteria were selected for further analysis using patient sera: (1) their hybridization signals were unambiguously detected in at least one of the samples being compared pairwise; (2) their transcript abundances (as judged by hybridization signal intensities) were three times or more higher in Siglec‐14, NTHi[+] than in Siglec‐14,NTHi[−]; and (3) their transcript abundances were either (3a) three times or more higher in Siglec‐14,NTHi[+] than in Siglec‐5,NTHi[+], or (3b) three times or more higher in Siglec‐14,NTHi[−] than in Siglec‐5,NTHi[−].

### Study subjects and protocol

We invited outpatients who visited the Respiratory Care Clinic at Nippon Medical School and were diagnosed as having COPD based on the Global Initiative for Chronic Obstructive Lung Disease (GOLD) criteria (Rabe et al. 2007) to participate in the study. The institutional review board at Nippon Medical School approved the protocols describing sample collection and related analyses in this study (approval numbers: 24‐09 and 24‐12). Written informed consent was obtained from each donor. Paired serum samples were collected from 71 COPD patients (discovery set, *n* = 39 and verification set, *n* = 32; no overlap between the two sets) who were seen both during stable and exacerbation states. Enrollment of 39 patients in the discovery set (November 2009–June 2011) preceded the initial analysis by multiplex bead array assay (analyzing seven parameters) described below, and that of 32 patients in the verification set (June 2011–September 2012) was after the initial analysis. Exacerbation was defined based on changes in baseline dyspnea, cough, and/or sputum exceeding normal day‐to‐day variations. Date of onset and presence of sputum purulence during exacerbation were based on the patient interview (diary and self‐assessment) during their monthly visits (Motegi et al. 2013). Stable state was defined as being “free from an exacerbation for at least 8 weeks.” Exacerbation sampling was only performed in patients who had not received systemic corticosteroids and/or antibiotics before their visit to the Respiratory Care Clinic.

### Measurements of clinical parameters

Postbronchodilator forced expiratory volume in 1 sec (FEV1), carbon monoxide‐diffusing capacity (diffusing capacity divided by alveolar volume, DLCO/VA), vital capacity (VC), and forced vital capacity (FVC) were measured according to the American Thoracic Society (1995) guidelines using a Pulmonary Function Test System (CHESTAC; CHEST M.I., Inc., Tokyo, Japan). Postbronchodilator FEV1 and VC, specified by the Japanese Respiratory Society (2001), were used as reference values.

We also performed helical high‐resolution computed tomography scans at 1.25 mm collimation, 0.8 sec scan time (rotation time), 120 kV, and 100–600 mA with a Light Speed Pro16 CT scanner (GE Co., Tokyo, Japan). The percentage of low attenuation area, reflecting the severity of emphysema, was calculated as described previously (Okazawa et al. 1996; Nakano et al. 2000; Orlandi et al. 2005).

### Quantification of serum proteins

Serum proteins were quantified using Procarta Cytokine Assay Kit, Human By Request (Panomics/Affymetrix, Santa Clara, CA), following standard procedures (Procarta Cytokine Assay Kit, User Manual Specifically for Serum and Plasma Samples) at Filgen (Nagoya, Japan). A custom 7‐plex assay (including CCL2, CCL20, CXCL1, soluble ICAM‐1, IL‐1*β*, IL‐8, and IL‐27) was used for the analysis of the discovery set (*n* = 39), and a custom duplex assay (including soluble ICAM‐1 and IL‐27) was used for the analysis of the verification set (*n* = 32). In each case, 25 *μ*L of serum was analyzed.

C‐reactive protein was measured at a clinical laboratory (BML Corporation, Tokyo, Japan) using N‐latex CRP II/*Cardio*Phase *hs*CRP (Siemens Health Care Diagnostics, Tokyo, Japan).

### Statistical analyses

**Figure 1.**
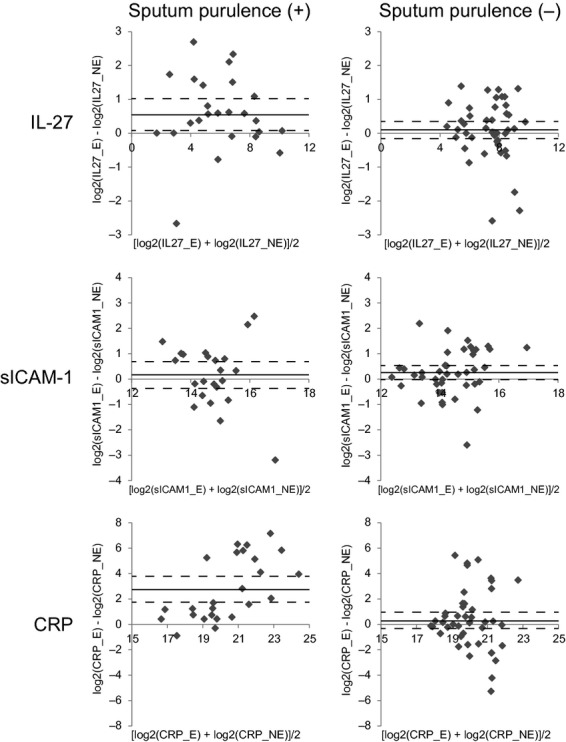
Changes of serum IL‐27, sICAM‐1, and C‐reactive protein (CRP) concentrations in association with exacerbation: stratification by the sputum purulence. The patient samples were stratified based on the presence (left column; *n* = 25) or absence (right column; *n* = 46) of sputum purulence during exacerbation. Each data point represents mean (*x*‐axis) and difference (*y*‐axis) of the analyte concentrations (pg/mL, represented in binary logarithm) in the paired patient sera collected during exacerbation and stable disease. Some data points (those that were above the quantifiable range) were omitted. Mean and 95% confidence intervals of *y* values are shown with solid and dotted lines, respectively.

**Figure 2.**
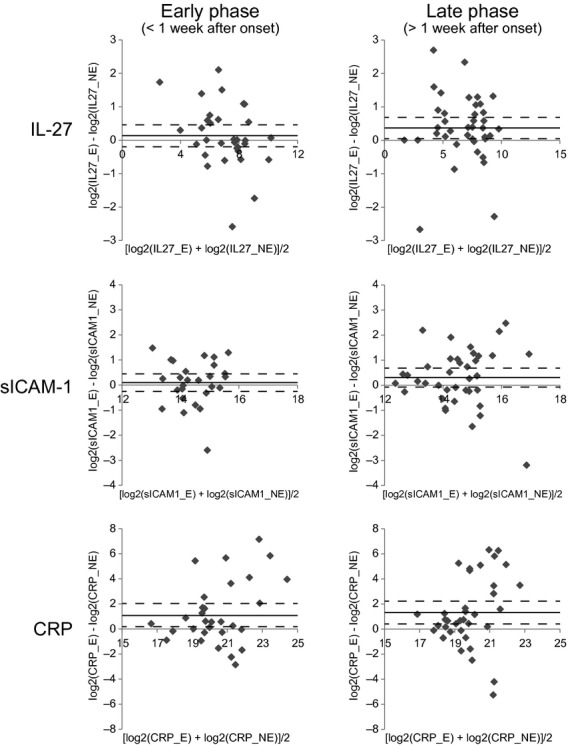
Changes of serum IL‐27, sICAM‐1, and C‐reactive protein (CRP) concentrations in association with exacerbation: stratification by the time of sampling after the onset of exacerbation episode. The patient samples were stratified based on the timing of the blood sampling, which was either within the first week (left column, “Early phase”; *n* = 32) or after (right column, “Late phase”; *n* = 39) the onset of exacerbation. Each data point represents mean (*x*‐axis) and difference (*y*‐axis) of the analyte concentrations (pg/mL, represented in binary logarithm) in the paired patient sera collected during exacerbation and stable disease. Some data points (those that were above the quantifiable range) were omitted. Mean and 95% confidence intervals of *y* values are shown with solid and dotted lines, respectively.

All statistical operations were performed using JMP genomics software version 5.1 (SAS Institute Inc., Cary, NC). Statistical tests employed in this study were as follows: comparison of patient characteristics between discovery and verification sets (Table 2): Fisher's exact test (gender, smoking status, and therapy), chi‐square test (GOLD stage), and Student's *t*‐test (other parameters); comparison of the serum protein concentrations between stable state and exacerbation (Table 3, Figs. 1, 2): one‐sided Wilcoxon matched‐pairs signed‐rank test. Thus, *P*‐values mentioned in Results were obtained by one‐sided Wilcoxon matched‐pairs signed‐rank test, unless otherwise stated.

## Results

### Identification of genes induced in Siglec‐14^+^ myeloid cells by NTHi stimulation

DNA microarray analysis was used to indentify genes whose expressions were induced in Siglec‐14^+^ myeloid cells by NTHi stimulation. We used THP‐1 human monoblastoid cell lines expressing either Siglec‐14 or Siglec‐5 (mimicking monocytes from wild‐type or homozygous *SIGLEC14*‐null individuals, respectively) as model myeloid cells, and screened for genes whose expressions were induced in Siglec‐14^+^ cells by stimulation with NTHi, and whose expressions were also (1) higher in stimulated Siglec‐14^+^ cells than in stimulated Siglec‐5^+^ cells; or (2) higher in nonstimulated Siglec‐14^+^ cells than in nonstimulated Siglec‐5^+^ cells.

Twelve genes showed one of these two patterns (Table 1). The products of nine of the twelve of these genes (*TNFAIP6*,* CCL20*,* IL1B*,* CXCL1*,* EBI3*,* ICAM1*,* TNFRSF9*,* CCL2*, and *IL8*) can be found in soluble forms in human bodily fluids. Most of them (8/12) are involved in the recruitment of leukocytes (*CCL20*,* CXCL1*,* CCL2*,* IL8*) or their functional modulation (*IL1B*,* EBI3*,* TNFRSF9*,* ICAM1*).

**Table 1.** Genes whose expression was induced in Siglec‐14^+^ THP‐1 human monoblastoid cell line by NTHi stimulation.

Gene symbol	Protein product (synonym)	Fold induction^1^	Criteria (3a)^2^	Criteria (3b)^2^
*TNFAIP6*	Tumor necrosis factor‐inducible gene 6 protein (TSG‐6)	11.0	No	Yes
*LOC285628*	–	10.1	No	Yes
*CCL20*	C‐C motif chemokine 20 (LARC, MIP‐3 *α*)	4.3	Yes	Yes
*IL1B*	Interleukin‐1 *β*	3.8	Yes	Yes
*RRAD*	GTP‐binding protein RAD	3.8	Yes	Yes
*CXCL1*	Growth‐regulated *α* protein (GRO‐*α*)	3.8	Yes	No
*EBI3*	Interleukin‐27 subunit *β* (EBV‐induced gene 3 protein)	3.6	Yes	Yes
*BIRC3*	Baculoviral IAP repeat‐containing protein 3 (RING finger protein 49)	3.5	No	Yes
*ICAM1*	Intercellular adhesion molecule 1 (CD54)	3.5	Yes	Yes
*CCL2*	C‐C motif chemokine 2 (MCP‐1)	3.4	Yes	Yes
*TNFRSF9*	Tumor necrosis factor receptor superfamily member 9 (CD137, 4‐1BB)	3.3	Yes	Yes
*IL8*	Interleukin‐8 (CXCL8)	3.3	Yes	Yes

^1^ The ratio of signal intensity in Siglec‐14/THP‐1 cells stimulated with NTHi as compared with that without stimulation.

^2^ See Materials and Methods section for details.

### Identification of IL‐27 as a possible COPD exacerbation marker

The products of seven of the twelve genes listed in Table 1, CCL2, CCL20, CXCL1, ICAM‐1, IL‐1*β*, IL‐8, and IL‐27 (a heterodimeric protein complex, of which *β* subunit is encoded by *EBI3*), were simultaneously quantified in serum using multiplex bead array assay.

To evaluate the utility of these candidate biomarkers in the diagnosis of COPD exacerbation, we measured their concentrations in paired serum samples from COPD patients collected during exacerbated and stable disease states. Patient characteristics are summarized in Table 2. We adopted a two‐tiered evaluation of marker candidates (discovery and verification steps) with independent sets of patient sera. One‐sided Wilcoxon matched‐pairs signed‐rank test was used to analyze the significance in concentration changes in marker candidates in sera during exacerbation.

**Table 2.** Patient characteristics.

Parameters	Discovery set (*n* = 39)	Verification set (*n* = 32)	*P* ^1^
Age	70.0 ± 7.5	72.2 ± 6.4	0.15
Gender (male/female)	39/0	27/5	0.01
Smoking status at the time of enrollment (current/ex)	7/32	2/30	0.17
GOLD stage (I/II/III/IV)	5/14/15/5	8/8/14/2	0.39
Pulmonary function tests			
%VC	86.6 ± 15.6	90.6 ± 22.1	0.39
FEV1	1.48 ± 0.66	1.38 ± 0.68	0.24
FEV1%	49.5 ± 12.4	48.4 ± 18.5	0.46
FEV1% predicted	52.8 ± 20.6	54.0 ± 23.8	0.86
%DLCO/VA	55.8 ± 23.5	57.5 ± 23.9	0.98
Computer tomography			
Low attenuation area (%)	36.0 ± 14.7	34.6 ± 15.6	0.91
Therapy (%)			
Long‐acting *β*2 agonist	87	91	1.00
Long‐acting muscarinic antagonist	82	94	0.29
Inhaled corticosteroid^2^	100	84	0.01
Systemic corticosteroid	0	0	–

%VC, actual/predicted vital capacity (VC) ratio, in percentage; FEV1, forced expiratory volume in 1 sec; FEV1%, FEV1/FVC ratio, in percentage; FEV1% predicted, actual/predicted FEV1 ratio, in percentage; %DLCO/VA, diffusing capacity for carbon monoxide/alveolar volume, in percentage.

Parameters are shown as mean ± standard deviation, unless indicated otherwise.

^1^ Comparison between discovery and verification sets. Fisher's exact test (gender, smoking status, and therapy), chi‐square test (GOLD stage), and Student's *t*‐test (other parameters) were used to compare the two groups.

^2^ Inhaled (low dose) corticosteroid is prescribed to most patients as a part of therapy during stable period, while systemic (high dose) corticosteroid may be prescribed/administered to patients only during exacerbation. Blood samples were not collected from those patients who were undergoing systemic corticosteroid treatment for the episode of exacerbation.

Of the seven marker candidates evaluated in the initial (discovery) phase, only soluble ICAM‐1 (sICAM‐1) and IL‐27 were found to be increased in sera from patients with exacerbated COPD (*P* < 0.05), though neither achieved statistical significance after Bonferroni correction (*P* < 0.05/7 = 0.0071; Table 3). The concentrations of these two marker candidates were measured in an independent set of paired sera in the second (verification) phase. Only IL‐27 increased in sera collected during exacerbation in this sample set, again with a nominal statistical significance (*P* = 0.0428; Table 3). As expected, analysis of the combined sample set (*n* = 71) improved the statistical significance (IL‐27: *P* = 0.0104; sICAM‐1: *P* = 0.0218; Table 3).

**Table 3.** Discovery and verification of systemic biomarker candidates of chronic obstructive pulmonary disease exacerbation.

Candidate biomarker	Discovery set (*n* = 39)			Verification set (*n* = 32)			Combined (*n* = 71)		
	Stable (pg/mL)	Ex (pg/mL)	*P* ^1^	Stable (pg/mL)	Ex (pg/mL)	*P* ^1^	Stable (pg/mL)	Ex (pg/mL)	*P* ^1^
CCL2	68.9 [43.8–105.2]	64.0 [43.8–117.1]	0.2019	–^2^	–	–	–	–	–
CCL20	×^3^	×	–	–	–	–	–	–	–
CXCL1	27.5 [19.4–39.5]	29.4 [21.2–37.1]	0.1675	–	–	–	–	–	–
sICAM	16081.3 [9809.3–24721.7]	17893.9 [12606.2–27916.9]	0.0170	28688.2 [22865.8–38989.6]	32171.9 [17540.3–46828.1]	0.2761	23240.9 [14788.8–32466.4]	22989.5 [14383.1–39428.9]	0.0218
IL‐1*β*	×	×	–	–	–	–	–	–	–
IL‐8	11.1 [4.6–29.1]	15.7 [7.3–33.4]	0.1693	–	–	–	–	–	–
IL‐27	267.2 [184.5–391]	290.0 [213.7–445.7]	0.0472	37.0 [18.3–58.7]	46.7 [32.7–76.0]	0.0428	159.5 [45.3–298.6]	200.8 [46.7–326.0]	0.0104

Values are reported as median [25th–75th percentiles].

^1^ One‐sided Wilcoxon matched‐pairs signed‐rank test.

^2^ Not tested.

^3^ Undetectable in most samples.

### Comparison of IL‐27 and sICAM‐1 with CRP

High‐sensitivity CRP is utilized in clinical practice as an auxiliary biomarker of COPD exacerbation to evaluate whether bacterial infection is involved in the exacerbation and to predict whether antibiotics may be useful for the treatment. We compared the properties of IL‐27 and sICAM‐1 with those of CRP. Samples were first categorized based on the presence or absence of sputum purulence during exacerbation (Fig. 1). CRP was elevated in the sera of patients whose sputum showed purulence, a sign of bacterially driven exacerbation (*P* < 0.0001), but it was not elevated in those without sputum purulence (*P* = 0.2450). This observation is consistent with CRP's utility as a biomarker of acute inflammation. IL‐27 was also elevated in the sera of patients with COPD exacerbation with sputum purulence (*P* = 0.0051), but not elevated in those without sputum purulence (*P* = 0.1355). However, sICAM‐1 was elevated in the sera of patients who were in an exacerbated state but had no sputum purulence (*P* = 0.0217), but not elevated in those who had sputum purulence (*P* = 0.2874).

The samples were then stratified by the timing of sample collection after the onset of exacerbation (Fig. 2). CRP concentrations were significantly higher in the sera collected within the first week of the onset of exacerbation (*P* = 0.0293) or later (*P* = 0.0014), while IL‐27 and sICAM‐1 concentrations were higher only in the sera collected more than 1 week after the onset of exacerbation (for IL‐27, within 1 week: *P* = 0.4695, after 1 week: *P* = 0.0010; for sICAM‐1, within 1 week: *P* = 0.1232, after 1 week: *P* = 0.0457).

## Discussion

In this study, we sought genes that were specifically induced in Siglec‐14^+^ myeloid cell line by NTHi stimulation to gain a better understanding of the mechanism underlying COPD exacerbation. We found seven potential candidates. We tested their utility as candidate biomarkers of COPD exacerbation and found IL‐27, which is also known to be involved in T‐cell differentiation and regulation, to be increased in the sera of COPD patient during exacerbation. This was particularly notable in patients with episodes accompanied with sputum purulence and those lasting more than a week.

There is a need to further investigate genes whose transcription levels are elevated specifically in Siglec‐14^+^ myeloid cells by NTHi stimulation. The chemokines CXCL1 and IL‐8, for example, bind to receptors on granulocytes (CXCR1 and CXCR2), while CCL2 and CCL20 bind to receptors on monocytes (CCR1 and CCR2) and dendritic cells (CCR6), respectively (Proudfoot 2002). These chemokines may coordinate the recruitment of innate immune cells and promote processes leading to COPD exacerbation. Other induced genes, for example, TSG‐6 and CD137, encode proteins that are thought to potentially be involved in the pathogenesis of asthma. TSG‐6, which is a hyaluronan‐binding protein encoded by *TNFAIP6*, has been found to be involved in eosinophilia and airway hyper‐responsiveness in a mouse model of asthma (Lauer et al. 2013; Swaidani et al. 2013). An antibody against CD137, which is a tumor necrosis factor receptor superfamily member encoded by *TNFRSF9*, has been reported to alleviate allergic asthma in a mouse model (Polte et al. 2006; Croft 2009). These genes/proteins may represent part of immune responses underlying asthma and COPD shared by both diseases. Therefore, it might be useful to study these proteins as possible etiological links to exacerbated COPD or as possible candidates as biomarkers of exacerbation.

We tested the possibility of using these seven gene products as systemic biomarkers of COPD exacerbation, and found IL‐27 levels to be elevated in the COPD patient sera during exacerbation. Serum IL‐27 was similar to CRP, a known biomarker of bacterial infection, in that levels of this gene product were increased during exacerbations associated with sputum purulence, itself a sign of bacterial involvement in exacerbation (Stockley et al. 2000). However, serum IL‐27 was significantly increased only in the sera collected in the second week or later after the onset of exacerbation, while elevations in CRP which were already evident in the first week of exacerbation, suggesting that IL‐27 might serve as a marker of prolonged exacerbation, rather than a marker of acute inflammation like CRP. These properties make IL‐27 an attractive candidate biomarker potentially useful in the assessment of etiology of COPD exacerbation and patient response to treatment. It would also be interesting to investigate whether there is an association between IL‐27 and the “gradual‐onset” type of exacerbations reported by Wedzicha's group (Aaron et al. 2012), as this type may overlap with the prolonged exacerbation in our study.

To the best of our knowledge, this study represents the first to report that the serum concentration of IL‐27 increases during COPD exacerbation. Higher baseline concentration of plasma IL‐27 has been reported in COPD patients (Cao et al. 2012), and a polymorphism in *IL27* gene (encoding IL‐27p28, i.e., IL‐27*α* subunit) has been associated with COPD (Huang et al. 2008). IL‐27, which is a heterodimeric cytokine consisting of IL‐27p28 and EBI3 subunits (also known as IL‐27*α* and *β*, respectively), is produced primarily by antigen‐presenting cells in response to microbial stimuli, and is involved primarily in the regulation of T‐cell differentiation and function (Wojno and Hunter 2012). Although T cells play pivotal roles in the pathogenesis of COPD (Cosio et al. 2009), the involvement of T cells in exacerbation has not been extensively documented, and the exact roles of T cells in exacerbation remain unclear (Tsoumakidou et al. 2005; Makris et al. 2008). This study suggests that T cells may play a role in COPD exacerbation. One possible mechanism through which the IL‐27 produced by myeloid cells affects COPD exacerbation is through Treg cells (Sakaguchi et al. 2008). Treg cell suppression by IL‐27 (Wojno and Hunter 2012) may lead to tissue damage by CD8^+^ T cells and/or the development of autoimmunity involving CD4^+^ T cells, and thus both can increase patient susceptibility toward exacerbation (Cosio et al. 2009).

It is important to note that we found sICAM‐1 to be increased in the sera of COPD patients without sputum purulence, as the increase in sICAM‐1 may represent a sign of viral exacerbation. Rhinovirus is a major causative agent of COPD exacerbation (Wedzicha 2001; Mallia et al. 2011). It utilizes membrane‐bound ICAM‐1 as host receptor for infection, while sICAM‐1 is known to neutralize rhinovirus (Marlin et al. 1990; Turner et al. 1999). Thus, further research into the functional relevance of ICAM‐1 in COPD exacerbation and its potential as a biomarker may be worthwhile. For example, it might be useful to study the utility of sICAM‐1 in combination of IL‐27 or other biomarkers of bacterial exacerbation (e.g., procalcitonin) (Stolz et al. 2007) in identifying the etiology of exacerbation and guiding the selection of treatment options.

Our study has some limitations. First, Siglec‐14 may not be involved in all episodes of exacerbation, and the candidate biomarkers of COPD exacerbation identified in this study may not be relevant to all cases. Nevertheless, our marker candidates are still likely relevant in a majority of exacerbations, given that homozygous *SIGLEC14*‐null patients tend to suffer less episodes of exacerbation (Angata et al. 2013), and that homozygous *SIGLEC14*‐null people are relative minority (especially among Europeans, in which the *SIGLEC14*‐null allele frequency is approximately 0.1) (Yamanaka et al. 2009). Second, IL‐27 may not be suitable for diagnosis using uniform “cutoff” value applicable to all patients. We attempted at assessing the specificity and sensitivity of IL‐27 for the diagnosis of exacerbation by drawing receiver‐operator curve, and found that area under the curve (AUC) for IL‐27 was 0.54, whereas that for CRP was 0.65 (data not shown). Given that AUC >0.8 is considered the standard for useful biomarker (Muller and Tamm 2006), this low AUC value for IL‐27 discourages its use as a stand‐alone biomarker of exacerbation. However, combination of multiple markers, or personalized approach (definition of “normal range” for each patient, rather than uniform cutoff value for all patients), may allow the utilization of IL‐27 in the future. Third, it is also important to point out that five of the seven gene products we chose as potential biomarker candidates were not found to be increased in sera collected during exacerbation or were not detected in most serum samples. This observation may be because the proinflammatory factors relevant to exacerbation are indeed mostly confined to local environments (Muller and Tamm 2006). Chemokines, which account for four of seven candidate markers in Table 2 (i.e., CCL2, CCL20, CXCL1, and IL‐8), are known to bind to heparan sulfate (Mortier et al. 2012), which is abundantly expressed not only on endothelia but also in the lung parenchyma (Smits et al. 2010). This fact may explain why many of our candidate markers were not found elevated in the patient sera during exacerbation. Such a constraint might pose a major obstacle to finding a systemic biomarker of COPD exacerbation.

In conclusion, this study, based on a human genetics and cell‐based model, provides valuable insight into the etiology of exacerbation and advances the field in its search for a biomarker of COPD exacerbation. IL‐27 may be one such promising systemic biomarker of this disease entity and deserves further research attention.

## Acknowledgments

We thank K. Fukumoto (Research Resource Center, RIKEN BSI) for DNA microarray analysis and M. Fujishiro (Nippon Medical School) for experimental assistance.

## Conflict of Interest

None declared.
